# Efficacy of nanomicelle curcumin, *Nigella sativa* oil, and their combination on bone turnover markers and their safety in postmenopausal women with primary osteoporosis and osteopenia: A triple‐blind randomized controlled trial

**DOI:** 10.1002/fsn3.2674

**Published:** 2021-11-30

**Authors:** Hanie Kheiridoost, Seyed Kazem Shakouri, Sara Shojaei‐Zarghani, Neda Dolatkhah, Azizeh Farshbaf‐Khalili

**Affiliations:** ^1^ Physical Medicine and Rehabilitation Research Center Aging Research Institute Tabriz University of Medical Sciences Tabriz Iran; ^2^ Gastroenterohepatology Research Center Shiraz University of Medical Sciences Shiraz Iran

**Keywords:** nanomicelle curcumin, *Nigella sativa* oil, osteopenia, osteoporosis, postmenopausal

## Abstract

Literature supports the potential effects of nanomicelle curcumin and *Nigella sativa* on the amelioration of osteoporosis, a health concern of postmenopausal women. This study aimed to evaluate the impacts of nanomicelle curcumin (CUR), *Nigella sativa* oil (NS), and their combination on bone turnover biomarkers and assess their safety. This triple‐blind randomized controlled trial was performed on 120 postmenopausal women aged 50–65 with primary osteoporosis or osteopenia. The subjects were randomly allocated to receive microcrystalline cellulose (placebo), 80 mg of CUR, 1000 mg of NS, or their combination (CUR‐NS) for 6 months. All patients were also treated with alendronate (70 mg) and calcium (500 mg), vitamin D (400 IU) supplements. The serum levels of alkaline phosphatase (ALP), osteocalcin (OC), and osteopontin (OP) were measured at the baseline and after the intervention. For safety assessment, the hepatic enzyme levels of aspartate transaminase and alanine transaminase as well as serum urea and creatinine were evaluated. ALP levels were significantly reduced in the NS (*p* = .029) and CUR‐NS (*p* = .015) groups compared with those in the placebo. After adjustment for the covariates, this effect was still significant in the CUR‐NS group (*p* = .004). The OC levels were decreased in the placebo, CUR, and NS groups, and the OP concentration also was attenuated in all groups through the trial. However, the intergroup differences were not significant for both biomarkers. Evaluating the key renal metabolites and hepatic enzyme levels indicated no toxicity of the administered doses. This study reveals the beneficial effects of CUR‐NS on the improvement of some bone turnover biomarkers. These compounds seem to be safe at the current dosage for supplementation in postmenopausal women.

## Graphical abstract

This study reveals the beneficial effects of nanomicelle curcumin and *Nigella sativa* oil on the improvement of some bone turnover biomarkers. These compounds seem to be safe at the current dosage for supplementation in postmenopausal women.

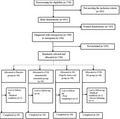

## 1 INTRODUCTION

Osteoporosis is the most common metabolic bone disorder associated with enhanced bone microarchitectural deterioration and the risk of fracture (Sözen et al., 2017). It is estimated that 9% to 38% of females and 2% to 8% of males are affected by osteoporosis in the developed countries ("Porter JL, Varacallo M. Osteoporosis. [Updated 2020 Nov 21]. In: StatPearls [Internet]. Treasure Island (FL): StatPearls Publishing; 2020 Jan‐. Available from: https://www.ncbi.nlm.nih.gov/books/NBK441901/,"). An imbalance between bone formation and bone resorption decreases bone mass and subsequently leads to osteoporosis. Primary osteoporosis is related to the deficiency of estrogen that is known as postmenopausal osteoporosis and also aging (Sözen et al., 2017). According to the international reference standard, osteoporosis in postmenopausal women is defined as a bone mineral density (BMD) of 2.5 standard deviation (SD) or more below the average value for healthy young female adults. Besides, the BMD T‐score between –1.0 and –2.5 SD is considered osteopenia (*World Health Organization. “WHO scientific group on the assessment of osteoporosis at primary health care level.” In Summary meeting report, vol. 5, pp. 5–7*. *2004*.). Multiple pharmacologic treatments are available for osteoporosis that mainly work through antiresorptive or anabolic mechanisms. Besides, many patients seek easily accessible herbal therapies due to the side effects of medical treatment (Ercan & Ibci, 2018). Therefore, evaluating the effectiveness of potential natural ingredients in the prevention and management of osteoporosis is an important goal of clinical research.

Curcumin (diferuloylmethane) is the chief constituent of turmeric spice (*Curcuma longa L*.) with several well‐known health‐promotional effects (Hewlings & Kalman, 2017; Rahmani et al., 2018). This natural polyphenol has been linked to the inhibition of oxidative stress (Li et al., 2020), osteoclastogenesis, and osteoclast proliferation (Bharti et al., 2004; Kim et al., 2011; Liang et al., 2020), and also the stimulation of osteoclasts apoptosis (Ozaki et al., 2000) and viability, growth, and differentiation of pre‐osteoblasts (Bukhari et al., 2019) in the in vitro and in vivo condition. *Nigella sativa* is another natural compound belonging to the Ranunculacea family with several health‐promotional effects (Tavakkoli et al., 2017) that reversed osteoporosis in the ovariectomized rats (Seif, 2014). There are also three clinical trials on the curcumin‐alendronate (Khanizadeh et al., 2018) and *Nigella sativa* oil (Hasani‐ranjbar et al., 2015; Valizadeh et al., 2015) effects on the BMD and bone turnover markers in a relatively small sample size of postmenopausal women with osteoporosis. Bone turnover markers are groups of proteins or protein‐derivative biochemical markers released during bone turnover by osteoblasts or osteoclasts. These biomarkers respond rapidly to the physiological changes in bone, so they are useful indicators for the diagnosis and therapeutic monitoring of osteoporosis (Burch et al., 2014; Greenblatt et al., 2017). Alkaline phosphatase (ALP) and osteocalcin (OC) are bone formation biomarkers, and osteopontin (OP) is a bone resorption indicator (Kuo & Chen, 2017).

Due to the limited studies and contradictory results, the present triple‐blind randomized controlled trial aimed to investigate the potential effects of nanomicelle curcumin, a more soluble form of curcumin, and *Nigella sativa* oil on bone turnover biomarkers, and their safety in postmenopausal women with primary osteoporosis and osteopenia. Moreover, combination therapy is usually more effective than the single‐agent treatment, and so, the effect of both agents was also investigated in the present study.

## 2 MATERIALS AND METHODS

### 2.1 Study design

The present factorial triple‐blind randomized controlled clinical trial (RCT) was carried out between August 2018 and October 2019, in the health centers of Tabriz, on 120 postmenopausal women with a new diagnosis of primary osteoporosis or osteopenia who met our inclusion criteria. This study is part of a megaproject approved by Tabriz University of Medical Sciences (Hemmati et al., 2021 ), which was also approved by the Ethics Committee of Tabriz University of Medical Sciences (IR.TBZMED.REC.1397.1032). The current study was registered in the Iranian Registry of Clinical Trials (IRCT20131009014957N4).

A blinded investigator, who was not involved in the clinical aspect of the research, randomly allocated the eligible women into four groups using a blocked randomization list stratified by the BMD T‐score (osteoporosis or osteopenia) in the Random Allocation Software (1:1:1:1). Allocation concealment was achieved using the sealed, opaque envelopes with consecutive numbering. The participants were assigned to receive the placebo of nanomicelle curcumin +placebo of *Nigella sativa* oil (the placebo group), 80 mg of nanomicelle curcumin +placebo of *Nigella sativa* oil (the CUR group), placebo of nanomicelle curcumin +1000 mg *Nigella sativa* oil (the NS group), or 80 mg of nanomicelle curcumin +1000 mg of *Nigella sativa* oil (the CUR‐NS group), once a day for 6 months. These doses were selected with the consideration of previous studies (Hasani‐ranjbar et al., 2015; Khanizadeh et al., 2018; Razmpoosh et al., 2020). At the baseline, a demographic characteristic questionnaire (including age, menopausal age, gravidity, parity, and duration of breastfeeding), a validated and reliable international physical activity questionnaire (IPAQ), and a three‐day food record were completed for the participants. Also, weight and height were measured, and body mass index (BMI) was calculated (weight (kg)/height (m)^2^) for all patients. Participants were asked to continue their routine eating habits and physical activity during the study and complete a checklist of daily supplement consumption. Besides, a sheet containing recommended guidelines of the National Institute of Arthritis and Musculoskeletal and Skin Diseases and also the amount of calcium and vitamin D in common foods was delivered to all the patients at the baseline. All participants received weekly alendronate tablets (70 mg) plus daily calcium‐vitamin D supplements (500 mg calcium +400 IU vitamin D) as standard treatment regimens. Researchers, participants, clinical and laboratory personnel, and statisticians were blinded to treatment allocation during the study period. Patients were followed up every 2 months for 6 months. Monthly telephonic reminders were given for medication compliance, and the compliance was also assessed at each visit by counting the remaining soft gelatin capsules with the patients and also completed checklists.

### 2.2 Participants

Postmenopausal women aged 50–65 with a low‐baseline BMD (T‐score ≤−1 at the lumbar spine or femoral neck), who had menopause for more than 1 year, lived in ([removed for Tabriz, Iran], and could take care of themselves and answer questions were selected to take part in the study. The exclusion criteria were T‐score ≤−4 in the lumbar spine or T‐score ≤−3.5 in the femur neck, history of pathological fractures, secondary osteoporosis, bone disorders other than osteoporosis, premature menopause, use of drugs affecting the bone metabolism (including intravenous bisphosphonate in the last 5 years, oral bisphosphonate in the prior 6 months, oral bisphosphonate for more than 3 years or more than 1 month between 6 and 12 months before the study, analogs of the parathyroid hormone or strontium during the previous 12 months, and hormonal drugs or corticosteroids during the study), kidney failure and the disorders, mental diseases, malignancies, gastric ulcer and gallstones, levels of 25‐(OH)‐vitamin D < 20 ng/ml, current hypercalcemia or hypocalcemia, coagulation disorders, use of anticoagulants, and history of allergic reactions to the examined supplements. The aims and scope of the investigation were orally explained to all women, and written informed consent was attained from each subject before study participation.

### 2.3 Characteristics of supplements

*Nanomicelle* curcumin soft gelatin capsules were obtained from Exir Nano Sina (Mashhad, Iran), which according to the drug catalog, each soft gel was standardized based on 80 mg of nanomicelle curcumin. The placebo capsules of curcumin were microcrystalline cellulose (MCC) with similar shape, color, smell, and size that also were purchased from this company.

*Nigella sativa* oil soft gelatin capsules were obtained from Barij Essence Pharmaceutical Co. (Kashan, Iran). Based on the company brochure, each capsule contained at least 6.5 mg of thymoquinone and 495–605 mg of linoleic acid. The placebo capsules of the *Nigella sativa* oil were MCC with similar shape, color, smell, and size that also were purchased from the mentioned company.

### 2.4 Blood sampling and turnover biomarker assessment

After 12 h of overnight fasting, 10 ml of venous blood samples were collected from each participant at the baseline and the end of the intervention. Serum samples were obtained by centrifugation at G‐force: 700 × g for 10 min at room temperature and stored rapidly at −80°C until biomarker assessments. The serum levels of OC and OP were measured using the enzyme‐linked immunosorbent assay (ELISA) kits (DuoSet ELISA Kit, R&D Systems; Catalog Numbers: DY1419‐05 and DY1433, respectively), according to the instructions of the company. The ALP, urea, creatinine, aspartate transaminase (AST), and alanine transaminase (ALT) concentrations were also determined spectrophotometrically (Mindray BS‐200 Chemistry Analyzer) using the Pars Azmoon™ detection kits (Pars Azmoon Co.) based on their specific kits' protocols.

### 2.5 Sample size and statistical analysis

The sample size was determined using G‐POWER software version 3.1.2, based on the previous study (BAYAT et al., 2008). Given α = 0.05, power = 95%, SD1 = SD2 = 0.178, and change in the mean of lumbar spine BMD = 0.104 (20% more than 0.92), the number of samples for each group was determined to be 26 that was increased to 30 to accommodate an expected 15% attrition rate. Data were analyzed according to the intention‐to‐treat analysis. The normality of the data was determined via skewness, kurtosis, and SD. The quantitative normally and non‐normally distributed variables were presented as mean ± SD and median (range), respectively. Differences between the four groups at the baseline or at the end of the intervention were assessed using the one‐way analysis of variance (ANOVA). The analysis of covariance (ANCOVA) was also performed for comparing the change in variables during the study between the groups by adjusting for baseline values and potential confounding factors. Fisher's least significant difference (LSD) post hoc test was carried out when significant differences were found. Besides, a paired‐sample *t*‐test was used to assess within‐group changes for all variables. *p*‐value less than .05 was considered significant. All data were analyzed using SPSS version 23 (SPSS).

## 3 RESULTS

**FIGURE 1:**
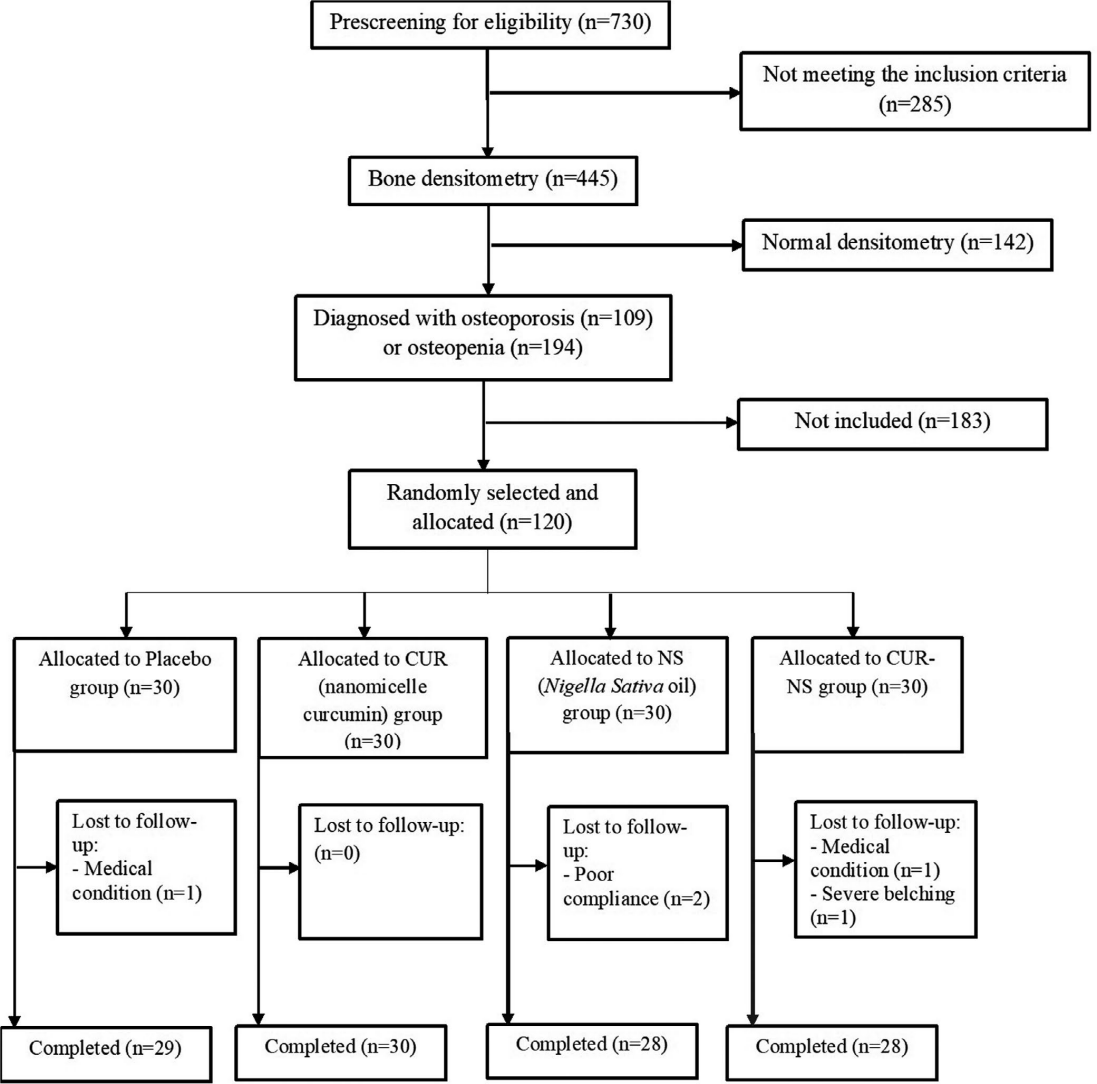
Flow chart of the study

The flowchart of the present study is shown in Figure 1. Of the 730 patients who were screened, 445 met our initial inclusion/exclusion criteria. After the dual‐energy X‐ray absorptiometry BMD measurement, 303 patients were diagnosed with osteopenia (*n* = 194) or osteoporosis (*n* = 109). Among them, a total of 120 potential participants were randomly selected and allocated into four equal groups. In the placebo, CUR, NS, and CUR‐NS groups, 18, 19, 18, and 18 patients had osteoporosis, respectively, and the others had osteopenia. One patient in the placebo group (*n* = 1 medical condition), two participants in the NS group (*n* = 2 poor compliance), and two patients in the CUR‐NS group (*n* = 1 medical condition, *n* = 1 severe belching) withdrew from the trial. Eventually, 115 patients, including 30 participants in the CUR group, 28 participants in each of the NS and CUR‐NS groups, and 29 participants in the placebo group, completed the follow‐up. Counting the remaining soft gelatin capsules confirmed that the participants had strictly adhered to the study protocol (>90% compliance).

Baseline characteristics of the patients in the different groups are presented in Table 1. Baseline total physical activity was significantly different between the studied groups (*p* =.012), and so, it was adjusted as a confounder in the analysis. There were no significant differences in the other baseline characteristics and also dietary intakes (data not shown) between the four groups.

**TABLE 1:** Baseline characteristics of the patients

Variable	Placebo group (*n* = 30)	CUR group (*n* = 30)	NS group (*n* = 30)	CUR‐NS group (*n* = 30)	*p*‐value
Age (year)	58.43 ± 3.41	58.00 ± 3.50	57.31 ± 4.37	57.43 ± 3.80	.640
Menopause age (year)	48.13 ± 3.73	48.76 ± 3.64	47.89 ± 4.40	48.60 ± 3.82	.816
Gravidity (number)	5.10 ± 2.11	3.85 ± 1.93	4.03 ± 2.11	4.96 ± 2.59	.074
Parity (number)	4.06 ± 1.43	3.35 ± 1.72	3.37 ± 1.61	4.17 ± 2.10	.149
Duration of lactation (month)	69.10 ± 42.05	59.42 ± 39.27	60.00 ± 33.57	75.59 ± 46.96	.409
Weight (kg)	70.10 ± 10.10	64.90 ± 8.94	67.47 ± 10.97	65.63 ± 8.26	.165
Height (cm)	156.08 ± 5.42	153.13 ± 6.95	155.48 ± 6.44	147.87 ± 30.02	.188
BMI (kg/m^2^)	28.77 ± 3.81	27.73 ± 3.81	27.89 ± 4.07	28.33 ± 3.68	.723
BMD (gr/cm³)					
Lumbar spine	0.76 ± 0.08	0.76 ± 0.09	0.76 ± 0.09	0.78 ± 0.09	.887
Femoral neck	0.77 ± 0.10	0.75 ± 0.08	0.77 ± 0.08	0.76 ± 0.11	.840
Total physical activity (MET‐min/week)	359.25 (4426.50)	792.00 (2493.00)	346.50 (4026.00)	749.25 (4666.50)	.**012**
Energy (kcal)	1923.79 (1000.71)	1853.13 (1485.01)	1855.76 (2140.54)	1820.28 (2030.07)	.531
Carbohydrate (g)	245.95 (249.21)	243.25 (282.33)	241.41 (487.30)	251.18 (272.08)	.992
Protein (g)	52.77 ± 13.60	53.02 ± 12.31	53.36 ± 14.63	55.65 ± 13.52	.835^†^
Total fat (g)	65.14 ± 17.00	65.60 ± 19.52	63.36 ± 18.29	63.82 ± 16.07	.956

Note

Total physical activity, energy, and carbohydrate were presented as median (range) and were statistically analyzed by the Kruskal‐Wallis test. Other data are expressed as mean ± SD and were analyzed using the one‐way ANOVA. *p*‐values of statistical significance (*p* < .05) are presented in bold.

Abbreviations: BMI, body mass index; BMD, bone mineral density; CUR, nanomicelle curcumin; NS, *Nigella sativa* oil.

Variations in total ALP, OC, and OP turnover biomarkers in all groups following the interventions are presented in Table 2. Regarding the serum levels of ALP, significant within‐group changes were observed in the NS (mean difference = −26.89, 95% CI: −46.14 to −7.65, *p*‐value = .008) and CUR‐NS (mean difference = −30.90, 95% CI: −50.28 to −11.53, *p*‐value = .003) groups. There was also a significant reduction in the mean differences of ALP in the NS (mean difference = −32.66, 95% CI: −61.92 to −3.40, *p*‐value = .029) and CUR‐NS (mean difference = −36.67, 95% CI: −66.19 to −7.15, *p*‐value = .015) groups compared with the placebo, according to the between‐group analysis. After adjusting for baseline values, total physical activity, age, BMI, gravida, menopausal age, and BMD of lumbar spine covariates, a significant reduction was only detected in the CUR‐NS group than in the placebo (mean difference = −36.74, 95% CI: −61.14 to –12.33, *p*‐value = .004) and CUR (mean difference = −26.77, 95% CI: −52.22 to –1.33, *p*‐value = .039) groups. The levels of OC also were significantly decreased in all groups, except the CUR‐NS. All four interventions also exerted a significant reduction in the OP levels. However, the between‐group differences in the OC or OP levels were insignificant at the end of the study.

**TABLE 2:** Comparison of the serum levels of bone turnover biomarkers at the baseline and after 6 months of intervention between the different groups

Variable	Placebo group (*n* = 29)	CUR group (*n* = 30)	NS group (*n* = 28)	CUR‐NS group (*n* = 28)	*p*‐value (between)^†^	Adjusted *p*‐value (between)^††^
ALP						
Baseline	186.78 ± 40.02	173.07 ± 46.96	198.57 ± 57.94	206.22 ± 52.79	.061	
After intervention	191.71 ± 62.56	172.41 ± 40.35	174.55 ± 41.78	171.08 ± 50.62	.358	
Mean difference (95% CI)	5.76 (−17.83 to 29.37)	−1.95 (−25.14 to 23.21)	−26.89 (−46.14 to −7.65)	−30.90 (−50.28 to −11.53)	.**023** ^a^	.**030** ^b^
***p*** ‐value (within)*	.621	.864	.**008**	.**003**		
Osteocalcin						
Baseline	27.57 ± 15.12	27.04 ± 12.28	27.84 ± 12.14	30.29 ± 18.13	.831	
After intervention	23.85 ± 12.57	23.15 ± 11.43	23.19 ± 10.07	24.96 ± 15.16	.940	
Mean difference (95%CI)	−4.36 (−7.33 to −1.39)	−3.89 (−7.41 to −0.37)	−5.03 (−9.49 to −0.57)	−5.04 (−10.79 to 0.72)	.975	.808
***p*** ‐value (within)*	.**006**	.**031**	.**029**	.084		
Osteopontin						
Baseline	31.29 ± 6.10	31.72 ± 4.18	30.53 ± 5.58	32.34 ± 5.77	.626	
After intervention	27.83 ± 7.93	25.01 ± 8.63	26.42 ± 7.51	27.61 ± 8.67	.541	
Mean difference (95%CI)	−3.86 (−6.44 to −1.28)	−6.50 (−9.70 to −3.30)	−4.10 (−6.50 to −1.70)	−4.54 (−7.46 to −1.61)	.505	.731
***p*** ‐value (within)*	.**005**	**<.001**	.**002**	.**004**		

Note

Data are expressed as mean ± standard deviation or mean difference (95%CI).

Abbreviations: ALP, alkaline phosphatase; CI, confidence interval; CUR, nanomicelle curcumin; NS, *Nigella sativa* oil.

*p‐value based on the paired‐samples *t*‐test. ^†^ *p*‐value based on the one‐way ANOVA. ^††^ *p*‐value based on the analysis of covariance (ANCOVA) adjusted for baseline measures, total physical activity, age, BMI, gravida, menopausal age, and BMD of lumbar spine. The LSD post hoc test was carried out when significant differences were found. *p*‐values of statistical significance (*p* < .05) are presented in bold.

^a^ Significant differences between the NS and placebo (*p* = .029), and the CUR‐NS and placebo (*p* = .015) groups.

^b^ Significant differences between the CUR‐NS group with the placebo (*p* = .004) and also CUR (*p* = .039) groups.

The safety of the administered supplements was assessed by the evaluation of renal and hepatic biomarkers, as shown in Table 3. Differences within and between the groups were statistically insignificant for urea at the end of the intervention. The baseline creatinine concentrations were significantly higher in the NS compared with those in the other groups. The levels of creatinine and ALT were significantly decreased in all four groups through the study period; however, the between‐group differences were insignificant at the end of the trial for both biomarkers. Within‐group differences of AST levels also indicated a significant reduction only in the NS group (*p*‐value = .019). No significant difference for AST was detected between the groups at the end of the trial (*p*‐value = .591).

**TABLE 3:** Comparison of the serum levels of renal and hepatic function biomarkers at the baseline and after 6 months of intervention between the different groups

Variable	Placebo group (*n* = 29)	CUR group (*n* = 30)	NS group (*n* = 28)	CUR‐NS group (*n* = 28)	*p*‐value (between)^†^	Adjusted *p*‐value (between)^††^
Urea						
Baseline	23.16 ± 5.14	23.38 ± 7.24	26.04 ± 6.43	24.83 ± 4.71	.301	
After intervention	26.64 ± 8.97	24.92 ± 6.38	28.23 ± 5.91	25.12 ± 7.15	.328	
Mean difference (95% CI)	2.76 (−1.79 to 7.31)	2.04 (−0.79 to 4.88)	2.64 (−0.46 to 5.74)	−0.15 (−4.23 to 3.93)	.638	.781
*p*‐value (within)*	.22	.148	.092	.939		
Creatinine						
Baseline	0.91 ± 0.12	0.92 ± 0.15	0.99 ± 0.15	0.88 ± 0.15	.**013** ^a^	
After intervention	0.66 ± 0.17	0.67 ± 0.28	0.77 ± 0.26	0.62 ± 0.21	.120	
Mean difference (95% CI)	−0.27 (−0.35 to −0.18)	−0.25 (−0.37 to −0.14)	−0.23 (−0.33 to −0.12)	−0.27 (−0.37 to −0.17)	.913	.104
*p*‐value (within)*	**<.001**	**<.001**	**<.001**	**<.001**		
AST						
Baseline	27.04 ± 8.72	28.65 ± 17.32	30.92 ± 12.28	26.87 ± 9.61	.668	
After intervention	25.25 ± 7.33	23.64 ± 6.39	24.08 ± 7.96	27.42 ± 20.29	.683	
Mean difference (95% CI)	−1.83 (−6.11 to 2.45)	−6.18 (−15.36 to 2.99)	−6.14 (−11.15 to −1.12)	−1.15 (−9.02 to 6.72)	.591	.899
*p*‐value (within)*	.379	.176	.**019**	.763		
ALT						
Baseline	15.61 ± 6.71	12.92 ± 7.22	15.17 ± 7.79	14.83 ± 7.71	.587	
After intervention	11.42 ± 4.73	9.20 ± 2.97	8.96 ± 4.19	9.71 ± 7.15	.307	
Mean difference (95% CI)	−4.33 (−8.02 to −0.65)	−3.59 (−6.09 to −1.09)	−5.62 (−8.81 to −2.43)	−4.95 (−8.75 to −1.15)	.810	0.845
*p*‐value (within)*	.**024**	.**007**	.**002**	.**013**		

Note

Data are expressed as mean ± standard deviation or mean difference (95%CI).

Abbreviations: ALT, alanine transaminase; AST, aspartate transaminase; CI, confidence interval; CUR, nanomicelle curcumin; NS, *Nigella sativa* oil.

*p‐value based on the paired‐samples *t*‐test. ^†^ *p*‐value based on the one‐way ANOVA. ^††^ *p*‐value based on the analysis of covariance (ANCOVA) adjusted for the baseline measures, total physical activity, age, BMI, gravida, menopausal age, and BMD of lumbar spine. The LSD post hoc test was carried out when significant differences were found. *p*‐values of statistical significance (*p* < .05) are presented in bold.

^a^ Significant differences between the NS and other groups.

Ninety‐four percent of the participants in the combined group, 90% in the NS and CUR groups, and 83% in the placebo group expressed that they were satisfied or completely satisfied with the medication. Thirteen percent of the participants in the placebo group, 10% in the NS and CUR groups, 7% in the combined group selected the option “neither satisfied nor dissatisfied.” Three percent in the placebo group were dissatisfied, and there was no significant difference between the groups (*p* = .689).

## 4 DISCUSSION

Bone is a dynamic tissue that is being broken down and rebuilt (turnover) in a balanced manner throughout the life span. Aging, menopause, and osteoporosis elevate the bone turnover (Garnero et al., 1996; Marie & Kassem, 2011). Menopause is linked to a 37%–52% and 79%–97% increased bone formation and resorption, respectively (Garnero et al., 1996). So, reduction in bone mass, deterioration of microarchitecture, and increase in the risk fracture are expected in such conditions (Garnero et al., 1996; Marie & Kassem, 2011; Shetty et al., 2016). Recently, biochemical markers of bone turnover, such as ALP, OC, and OP, are linked to low‐baseline BMD and also are considered to be predictors of fracture, independent of BMD (Chen et al., 2018; Fodor et al., 2013; Nakamura et al., 2017; Shetty et al., 2016).

ALP is one of the most frequently examined bone turnover biomarkers in clinical and research tests. This protein is a ubiquitous and membrane‐bound phosphorylating enzyme with many isozyme forms in the intestine, placenta, liver, and bone. Bone‐specific ALP (BALP) attaches to the osteoblastic cell membrane and releases in the serum in a small amount (Mukaiyama et al., 2015; Shetty et al., 2016; Tariq et al., 2019). The serum concentration of total ALP and BALP rises in postmenopausal women due to increased bone remodeling (Mukaiyama et al., 2015). In the present RCT, ALP levels significantly were reduced in the NS and CUR‐NS groups according to within‐ and between‐group comparisons. This effect remained significant only in the CUR‐NS group after adjusting for multiple covariates. This result is partly in line with a previous meta‐analysis that demonstrated the effect of *Nigella sativa* on reducing the total ALP levels in 710 participants with different health status (weighted mean difference (WMD) = −10.825; 95% CI: −19.658, −1.992 U/L; *p*‐value = .016). In this study, a subgroup analysis based on the dosage of the *Nigella sativa* supplement revealed that this beneficial effect was stronger at the daily dose of 1100–2000 mg rather than the lower doses (Razmpoosh et al., 2020). Some studies also demonstrated the antiosteoporotic effects of *Nigella sativa* or its most abundant ingredient, thymoquinone, on the animal models of postmenopausal or diabetes‐induced osteoporosis (Seif, 2014; Shuid et al., 2012). On the contrary, no effect of 3 ml of *Nigella sativa* oil supplementation for 3 months on the serum levels of BALP and ALP in postmenopausal women with osteoporosis was reported in a pilot RCT (sample size in the NG group = 5) (Valizadeh et al., 2015). Moreover, another study by Hasani‐ranjbar et al. indicated no change in the serum levels of BALP after treatment with 600 mg of *Nigella sativa* twice a day (Hasani‐ranjbar et al., 2015). The difference examined the doses and the follow‐up durations of the *Nigella sativa* supplementation in various studies that can partly explain these conflicting and ambiguous results. Besides, regarding the curcumin effects, a previous study revealed a significant reduction in the serum BALP and an increase in BMD in the postmenopausal women with osteoporosis treated with 110 mg/day of curcumin +5 mg/day of alendronate +1000–1500 mg/day of calcium for 12 months compared with the other groups receiving alendronate +calcium or the calcium supplements alone (Khanizadeh et al., 2018). In another study, the curcumin supplementation at the dose of 110 mg/kg for 6 months reduced BALP levels and the risk of osteoporosis and also increased the mean BMD of the femoral neck and hip in patients with the spinal cord injuries (Hatefi et al., 2018). The anti‐inflammatory, anti‐oxidative, and estrogenic activities of *Nigella sativa* and curcumin may play undeniable roles in the primary osteoporosis treatment because this bone disorder has been linked to inflammation, oxidative stress, and estrogen deficiency (Bachmeier et al., 2010; Menon & Sudheer, 2007; Parhizkar et al., 2016; Shuid et al., 2012; Weitzmann & Pacifici, 2006).

The effects of nanomicelle curcumin, *Nigella sativa* oil, and their combination on the serum OC and OP also were investigated in the present RCT. The results showed the reduction in OC levels in all groups, except the combination treatment, and the attenuation of OP concentrations in all four studied groups. However, intergroup differences were not significant for both markers. Similarly, two previous RCTs demonstrated no impact of the *Nigella sativa* therapy on the OC levels in the postmenopausal osteoporotic women (Hasani‐ranjbar et al., 2015; Valizadeh et al., 2015). However, two previous studies showed contradictory results of *curcumin* on the increase and decrease in the OC concentration in postmenopausal osteoporotic and spinal cord injury–induced osteoporosis conditions, respectively (Hatefi et al., 2018; Khanizadeh et al., 2018). The differences in the doses, follow‐up durations, and health status of the participant can explain these conflicting results. There are no previous studies on the effects of curcumin or *Nigella sativa* on the OP levels of postmenopausal osteoporotic women. In the current RCT, the OC and OP levels also were reduced in the placebo group during the follow‐up period. The administration of alendronate tablets and calcium‐vitamin D supplements, as standard therapeutical strategies, as well as providing related dietary advice to all participants, even the control group, may explain the observed effect.

In the present study, we also assessed the safety of the interventions by evaluating the key renal metabolites and hepatic enzyme levels, including urea, creatinine, AST, and ALT, which found no significant intergroup differences in all biomarkers. So, the examined doses of nanomicelle curcumin and *Nigella sativa* oil may be safe for postmenopausal women. Similarly, previous studies also found no toxic effects of curcumin administration on humans even at a dose of 8 g/day (Chainani‐Wu, 2003). Moreover, *Nigella sativa* oil is considered “generally recognized as safe” (GRAS) by the U.S. Food and Drug Administration, and the safety of its long‐term use is reported in various scientific studies (Goyal et al., 2017; Tavakkoli et al., 2017; Zaoui et al., 2002).

Curcumin is a compound with relatively low solubility in water and its oral bioavailability is inadequate, which limit its usage in clinical trials. However, it is reported that the encapsulation of curcumin in nanomicelles can overcome these obstacles (Hatamipour et al., 2019; Rahimi et al., 2016). In the current study, for the first time, we investigated the effects of CUR, NS, and their combination on bone turnover biomarkers. The present RCT had some limitations, notably the inability to assess other bone turnover biomarkers, and also the anti‐inflammatory and antioxidant activities of the interventions. It is recommended to conduct the future trials to investigate the impact of the nanomicelle curcumin, *Nigella sativa*, and their combination on other bone turnover biomarkers especially C‐terminal telopeptide of type 1 collagen (CTX) and procollagen type 1 aminoterminal propeptide (P1NP) as reference bone resorption and formation biomarkers, respectively, in osteoporosis (Vasikaran et al., 2011). Further study on the effects of these compounds on osteopenia and osteoporosis, separately, or on secondary osteoporosis is recommended. Moreover, it is noteworthy that osteoporosis was diagnosed in all women studied at the baseline, and we could not morally deprive people of routine medication. Considering the results of this study, it will probably be possible to compare the combination of nanomicelle curcumin and *Nigella sativa* with alendronate in the future studies considering the necessary follow‐ups and investigations.

## 5 CONCLUSION

This study revealed the beneficial effects of CUR‐NS on the improvement of some bone turnover biomarkers. A stronger surrogate for bone strength, such as BMD, needs to be further explored in women under the CUR‐NS supplementation. Both nanomicelle curcumin and *Nigella sativa* oil seem to be safe in the used doses for postmenopausal women. However, more studies are needed to establish the outcomes of the present trial and shed light on the related mechanisms.

## CONFLICT OF INTEREST

The authors declare that they do not have any conflict of interest.

## AUTHOR CONTRIBUTIONS

**Hanie Kheiridoost:** Conceptualization (equal); Data curation (equal); Investigation (equal); Writing–original draft (equal). **Seyed Kazem Shakouri:** Conceptualization (equal); Formal analysis (equal); Funding acquisition (equal); Project administration (equal); Writing–review & editing (equal). **Sara Shojaei‐Zarghani:** Investigation (equal); Visualization (equal); Writing–original draft (equal). **Azizeh Farshbaf‐Khalili:** Conceptualization (equal); Data curation (equal); Formal analysis (equal); Investigation (equal); Project administration (equal); Visualization (equal); Writing–review & editing (equal).

## ETHICAL APPROVAL

This study was approved by the Ethics Committee of Tabriz University of Medical Sciences (IR.TBZMED.REC.1397.1032). This work complies with the CONSORT guideline.

## INFORMED CONSENT

Written informed consent was obtained from all study participants.

## Data Availability

The data are available from the responsible author upon reasonable request.
